# VISION – an open-source software for automated multi-dimensional image analysis of cellular biophysics

**DOI:** 10.1242/jcs.262166

**Published:** 2024-10-23

**Authors:** Florian Weber, Sofiia Iskrak, Franziska Ragaller, Jan Schlegel, Birgit Plochberger, Erdinc Sezgin, Luca A. Andronico

**Affiliations:** ^1^ Science for Life Laboratory, Department of Women's and Children's Health, Karolinska Institutet, 17165 Solna, Sweden; ^2^ Department Medical Engineering, University of Applied Sciences Upper Austria, 4020 Linz, Austria; ^3^ LBG Ludwig Boltzmann Institute for Traumatology, Nanoscopy, 1200 Vienna, Austria

**Keywords:** Multi-dimension microscopy, Spectral Imaging, Image analysis, Biophysical properties, Open source, Python

## Abstract

Environment-sensitive probes are frequently used in spectral and multi-channel microscopy to study alterations in cell homeostasis. However, the few open-source packages available for processing of spectral images are limited in scope. Here, we present VISION, a stand-alone software based on Python for spectral analysis with improved applicability. In addition to classical intensity-based analysis, our software can batch-process multidimensional images with an advanced single-cell segmentation capability and apply user-defined mathematical operations on spectra to calculate biophysical and metabolic parameters of single cells. VISION allows for 3D and temporal mapping of properties such as membrane fluidity and mitochondrial potential. We demonstrate the broad applicability of VISION by applying it to study the effect of various drugs on cellular biophysical properties. the correlation between membrane fluidity and mitochondrial potential, protein distribution in cell–cell contacts and properties of nanodomains in cell-derived vesicles. Together with the code, we provide a graphical user interface for easy adoption.

## Teaser

**Summary:** VISION, an open-source software, enables high throughput and correlative analysis of cellular biophysical properties.

## INTRODUCTION

Biophysics aims at understanding the correlations between physical changes in cell and biological processes (Horwitz, 2016). For example, researchers have demonstrated how remodelling of membrane biophysical properties, including polarity, tension, fluidity and depolarization, can influence processes such as cell proliferation and migration (Atilla-Gokcumen et al., 2014; Braig et al., 2015; Edmond et al., 2015; Kunduri et al., 2022) and the immune response (Rentero et al., 2008; Rudd-Schmidt et al., 2019; Tello-Lafoz et al., 2021). Recent advances in technology for high-throughput characterization of cell biophysics, have also highlighted the importance of such biophysical remodelling in diseases (Andronico et al., 2024 preprint). Among others, environment-sensitive probes are often used to investigate alterations in cellular biophysics. These probes undergo changes in their photophysical properties (e.g. emission intensity and/or spectrum, and fluorescence lifetime) in response to changes in the surrounding lipid composition or bilayer hydration (García-Calvo et al., 2022; Ragaller et al., 2022, 2024). Typical examples of membrane-intercalating dyes, which undergo an emission red-shifting upon increase in membrane fluidity are LAURDAN, NR12A/S and Pro12A (Amaro et al., 2017; Danylchuk et al., 2020; Niko and Klymchenko, 2021; Parasassi et al., 1990). These probes can be chemically functionalized to target either the plasma membrane (PM) or the lipid bilayer of different subcellular compartments (Klymchenko, 2023). Thus, fluorescence microscopy is often used to study 2D and 3D dynamic remodelling of cell biophysics, specifically under spectral or multi-channel modality. By acquiring a greater extent of the emission spectrum, the resolution and sensitivity of measurements can be enhanced (Fereidouni et al., 2012; Sezgin et al., 2015). Then, the image analysis usually requires some form of mathematical operations to derive a practical readout of the biophysical properties under investigation. This could be as simple as calculating the ratio between two intensities (e.g. to measure mitochondrial membrane depolarization via JC-1 dye) (Sivandzade et al., 2019), or it might involve a more elaborated equation or fitting, for instance as for the calculation of the generalized polarization (GP), which is used to determine membrane fluidity (Parasassi et al., 1990). Several image analysis packages have been developed to analyse microscopy images and to measure properties, such as the morphology or number of particles for an object. Some software rely on fluorescence intensity values for segmentation, for instance SimFCS and CellProfiler (Rossow et al., 2010; Stirling et al., 2021), whereas others follow the phasor analysis approach, specifically on spectral images [e.g. PhasorPy and the Spectral Phasor plugin for Fiji (PhasorPy documentation, see https://www.phasorpy.org/stable/)]. Often, software are applicable either to certain geometries, such as spherical vesicles (van Buren et al., 2022; Stirling et al., 2021), or specialized in analysing specific subcellular compartments, such as the PM or the cell nucleus (Barry et al., 2022). The options decrease even further when one needs to analyse cellular biophysical properties, which requires mathematical operations. Here, we introduce a new tool, called VISION, for analysis of 2D or 3D multi-channel and spectral images, implemented in Python and featuring a user-friendly graphical interface (GUI; Figs. S4–S7). VISION software, the core Python code, user guide, troubleshooting and tutorials can be freely downloaded from the GitHub repository at https://github.com/biosciflo/VISION. In addition to canonical intensity-based analysis, our software enables profiling of several cell biophysical parameters (i.e. membrane fluidity, mitochondria membrane potential etc.) in an automated fashion and from multiple image formats. The possibility to calculate multiple biophysical readouts is achieved through the ability to perform customizable mathematical operations on the different acquired channels. Some of the key features are independent masking of the PM and cytosol, whole-image versus single-object analysis, morphological characterization and high-resolution membrane profiling as well as cell linearization. We demonstrate the advantages of our software in four different biological scenarios: (1) characterization of phase stability in cell-derived vesicles; (2) correlation between mitochondria health and fluidity of the plasma membrane under pathological settings; (3) mapping of the protein accumulation and clustering in reconstituted immune synapses, and (4) profiling of the membrane fluidity in phase-separated vesicles at submicrometre resolution. We believe our software represents a complementary tool for researchers who are interested in investigating spatiotemporal aspects of biophysical and metabolic remodelling caused by cellular processes and external stimuli.

## RESULTS

### Software overview

**Fig. 1.**
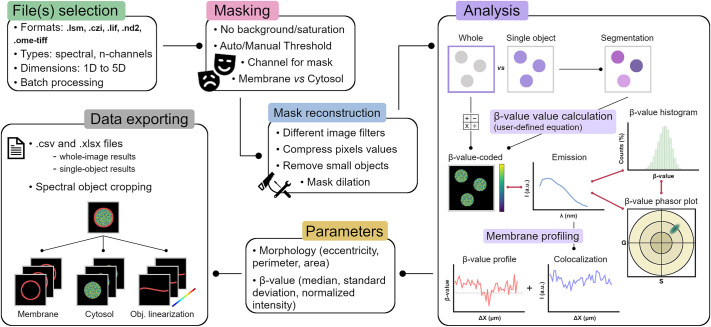
**Illustration of the VISION general workflow.** The different boxes show the main features of the VISION software. ΔX refers to the lateral displacement during profiling of the β-value (i.e. the biophysical parameter under investigation) along the vesicle's membrane.

Most of the functions utilized in our software are sourced from common open-source packages (i.e. scipy, https://scipy.org/; scikit-learn, https://scikit-learn.org/stable/; matplotlib, https://matplotlib.org/), whereas others were developed by us to perform specific tasks during the analysis. VISION is highly versatile in terms of input image types that can be analysed. It supports various file formats (Fig. 1) and it can handle multi-dimensional images, including *t*-stack, *z*-stack and *t-z*-stack formats. Additionally, VISION offers batch processing capabilities. The software is designed to take advantage of the enhanced detail in measuring biophysical properties in cells, which derives from working with spectral images (Aron et al., 2017). Therefore, it can process multi-dimensional images with one and up to *n* λ-channels. A key aspect of analysing microscopy images is the creation of a proper mask. In this regard, VISION offers users significant flexibility, enabling independent masking of the plasma membrane and cytosolic parts, as well as performing mask morphological reconstruction after thresholding. After the mask has been created, the user can choose which biophysical parameter (which we refer to as a generic β-value) to calculate (via a customized equation), and whether to perform the analysis on the image as a whole or on individual objects. Thus, several outputs are generated such as: (1) the reconstructed emission spectrum, (2) the β-value colour-coded image, (3) a histogram of the β-value distribution and (4) the phasor plot of β-value distribution (Fereidouni et al., 2012). The single-object mode allows for the extraction of additional descriptive statistical and morphological information on individual objects. It also enables the export of a spectrally reconstructed image of either membrane/cytosol for the individual object or the linearized object. Finally, all datasets are saved and exported as both a .csv and a .xlsx file to enable further downstream analysis. With its numerous implemented features, our software can be utilized for quantitative analysis of a variety of different samples, such as single particles, vesicles or cells.

### Masking

**Fig. 2.**
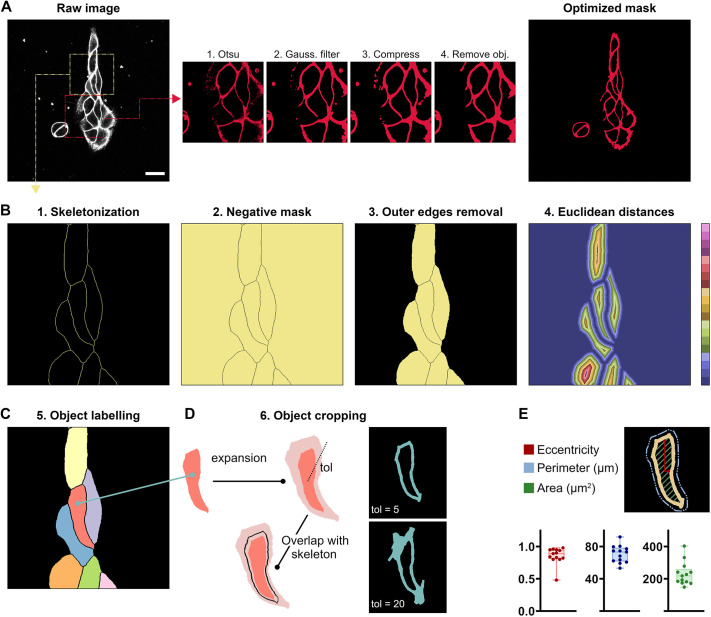
**Working principles of image segmentation.** (A) Optimization of the mask for the analysis of a parameter on cells. Starting from the raw image, different independent operations can be performed on the preliminary mask for optimization. (B,C) Working principle for the image segmentation. Steps 1 to 5 show the operations performed on the optimized mask to obtain a final image of individually labelled objects. (D) Consecutive steps for the cropping of individual objects. Depending on the tolerance parameter (tol) the user can expand or reduce the cropped object. For the plots, the box represents the 25–75th percentiles, and the median is indicated. The whiskers show the range. Each data point corresponds to an individual cell in Fig. 2A. (E) Object-specific morphological parameters calculated by the software. Scale bar: 5 µm.

In assessing the biophysical properties, the incorporation of noisy pixels (i.e. background fluctuations) in the calculation of the β-value could potentially have a pronounced impact on the measurement accuracy. These pixels introduce uncertainty, which propagates and increases due to the mathematical operations typically performed to derive the β-value. For this reason, the software offers the capability to specify a particular signal-to-noise ratio for filtering out background pixels before image thresholding, and it autonomously eliminates saturated pixels. Furthermore, the user can apply different filters (median, mean, gaussian etc.) and, if working with multi-channels images, specify on which channel or combined channels to perform the thresholding. The analysis of individual objects by VISION relies on creating a skeleton from the thresholded image. Thus, for membranes, it is fundamental to obtain a mask where the membrane is a continuum of pixels. This can be achieved by performing different implemented operations on the mask. For instance, the insets in Fig. 2A, compare the results obtained when (1) applying only an automatic thresholding via Otsu algorithm, (2) applying a Gaussian filter before the automatic thresholding and (3) compressing the pixel values in between the Gaussian filter and the thresholding via Otsu. Specifically, the use of our custom-defined function for pixel compression (Fig. S1) allows thresholding of areas with different brightness via the Otsu algorithm, thus preserving the automation of thresholding during batch processing. This approach greatly simplifies the masking procedure compared to that seen in other software where multiple masks are required to deal with images with heterogenous brightness (Stirling et al., 2021). The user can also perform morphological reconstruction of the mask to remove small clusters before the segmentation step (Fig. 2A, inset 4) and decide the channel (for spectral images) to use for image thresholding. Finally, within the GUI, users have the option to define either global or slice-specific parameters (e.g. for *t*-stacks) for masking before initiating batch analysis.

### β-value calculation

Once the mask has been optimized, the software provides the option to select up to four channels for calculating the biophysical parameter (β-value) of interest. Additionally, users can define a mathematical equation for determining the β-value. This is independent for the membrane and the cytosol calculation, thus allowing for simultaneous characterization of multiple biophysical or metabolic properties and correlative analysis. As a result, it generates a reconstructed pseudo-coloured image displaying the pixel-wise β-values for membrane and cytosol parts individually. The equation supports all the main algebraic operations, and pixels that return erroneous values are automatically removed from the analysis. Statistical descriptors for the β-values (i.e. median, standard deviation) are generated from either the whole image or individual objects, depending on the mode of operation.

### Segmentation

Single-object analysis from microscopy images usually requires two distinct steps: image segmentation (especially when working with clustered objects such as adherent cells) followed by the object identification or labelling. Available software uses either morphology-related (van Buren et al., 2022) or AI-based algorithms (Berg et al., 2019; Körber, 2022) to perform object detection. In VISION, object detection is accomplished through a six-step algorithmic process (Fig. 2B–D). This process can be applied to images containing a mixture of isolated and clustered objects, with shapes ranging from simpler to highly irregular. First, a raw skeleton is derived from the optimized mask and subjected to a customized function for removal of side branches; the skeletonized mask is converted into a negative mask where the inner part of the object is filled, and the outer edges are removed. This operation ensures that objects sitting at the image edges (i.e. whose membrane is cropped) will be ignored during the analysis. The mask of filled objects is then converted into the corresponding mask of pixel-to-pixel Euclidean distances. At this stage, a user-defined tolerance value (*tol_0_*) will be used to threshold only those distances above *tol_0_*, thereby causing a greater or smaller retraction from the skeleton of the vesicle. Subsequently, shrunken vesicles will be progressively labelled after undergoing pixel clustering via the DBSCAN algorithm. DBSCAN is a density-based algorithm that performs effectively on arbitrarily shaped vesicles and without the need to predefine the number of clusters (Martin Ester, 1996). The last step of image segmentation represents the cropping of individual vesicles via consecutive expansion of the shrunk vesicles (according to a second user-define tolerance value *tol*) and overlap with the general skeleton. The software returns a set of morphological parameters for each individual vesicle, such as eccentricity, perimeter and area for vesicles (Fig. 2D,E), which are calculated using modules from the open-source scikit-learn package (Pedregosa et al., 2011).

### PM profiling

**Fig. 3.**
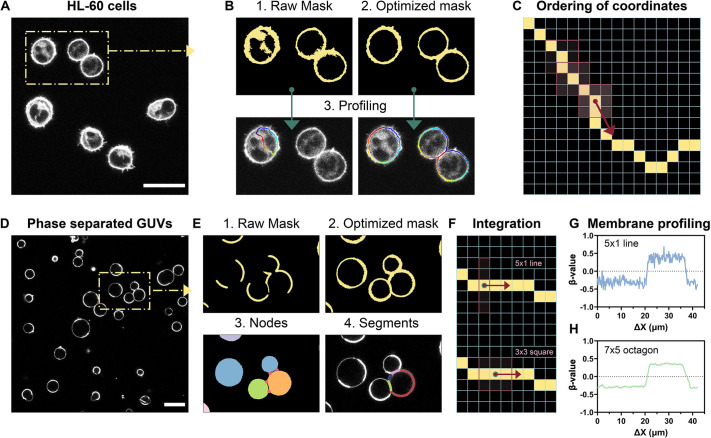
**Profiling of membranes.** (A) Raw image of human leukaemia suspension cell lines (HL-60) stained with an environment-sensitive probe (Pro12A) used for measurement of membrane fluidity. (B) The effect of mask optimization on the profiling of the plasma membrane. The raw mask is obtained by applying an automatic threshold via the Otsu algorithm, whereas the optimized mask is derived by choosing a channel with low cytosolic background for thresholding and applying the function for pixel compression, to obtain a continuous membrane. (C) Schematic for the ordering of the pixel coordinates from the 1-pixel wide line used for the membrane profiling. The line (in yellow) is centred onto the plasma membrane, and a 3×3 pixel square box is slid along the line. (D) Raw image of phase-separated GUVs stained with an environment-sensitive probe (NR12S) used for measurement of membrane fluidity. (E) The effect of mask optimization for membrane profiling and segment detection in clustered vesicles. The raw mask is obtained by applying an automatic threshold via the Otsu algorithm. Then, the optimized mask is derived by performing the following steps: (1) select a channel for thresholding in which the two phases have similar brightness; (2) applying compress pixel values according to the dedicated function and (3) performing dilation of the binary mask to obtain a continuous membrane. On the optimized mask, the software detects nodes in clustered vesicles and, if the profiling mode is activated, it profiles individual segments (4). (F) Schematic for the signal integration during membrane profiling (line versus square). (G,H) The effect of choosing a different integrating element on profiled curves for β-value (i.e. parameter under investigation). The larger the area of the integrating element the smother the profiled curve. Scale bars: 25 µm. ΔX represents the lateral displacement along the membrane of the vesicle in µm.

A key feature of the VISION software is the possibility to profile the membrane and track fluctuations of the β-value and/or channel intensities at a resolution that is limited only by the pixel size. This characteristic is useful, for instance, when studying the dynamic rearrangement of biophysical properties at cell–cell contacts, to monitor protein accumulation in phase-separated lipid bilayers or protein clustering at the membrane sub-compartments (Lamerton et al., 2021). Fig. 3 shows the principles behind the module for membrane profiling applied to two different scenarios: suspension cells (HL-60, Fig. 3A,B) and phase-separated giant unilamellar vesicles (GUVs, Fig. 3D,E), both stained with an environment-sensitive probe for membrane fluidity. Our approach relies on creating a 1-pixel wide guiding line centred onto the membrane that will be used to move a 2D integrating element unidirectionally along the bilayer for signal integration. Therefore, it is important to avoid any contribution from the cytosol when analysing cells (Fig. 3B), or to ensure continuity of the masked membrane when analysing phase-separated synthetic vesicles with areas of different brightness (Fig. 3E) during the masking step. Once the mask is created, it will undergo skeletonization, morphological reconstruction of the skeleton and unidirectional re-ordering of the coordinates of the pixels comprising the line, via a customized function (Fig. 3C). In case of clustered objects, the software will automatically detect the intersection nodes and identify the different segments which constitute membrane of the clustered object. Finally, a moving average filter shaped according to user-defined parameters will traverse along the ordered skeleton (Fig. 3F) and generate trajectory curves reporting the average intensity or β-value (Fig. 3G,H, ΔX refers to the lateral displacement of the moving average filter in µm). By adjusting the shape and size of the moving average filter, users can obtain smoother trajectories if desired (Fig. 3G versus H), although at the cost of spatial resolution. Common methods for membrane profiling involve calculating the integrated signal from segments of the membrane by slicing it into portions of equal angular amplitude (van Buren et al., 2022). Although this method works for spherical objects, it might yield inaccurate results on cells with irregular shapes. By contrast, our approach ensures the same integrating area, thus leading to a greater accuracy even when working in the presences of membrane irregularities, such as budding, invagination etc. The GUI also offers the option to perform a recentring of the guiding line onto the membrane (useful when analysing membranes that present clusters or aggregated proteins) (Fig. S2) and the colocalization between the biophysical parameter of choice (β-value) and the signal from a third channel of interest.

### Data exporting

For each analysed image, VISION generates both a .csv and an .xlsx files that are automatically updated after analysis, depending on the specific analysis performed. Column headers are provided to facilitate navigation of the file content. Descriptive statistics on the β-value, the emission spectrum and the β-value histogram are generated from the whole image as well as for individual object detected (when performing image segmentation). Furthermore, when the option for membrane profiling is selected, the object-specific trajectories for channel intensities, β-value and additional channel for colocalization are saved, together with the information regarding the number of segments and corresponding length per object (in case of clustered objects). Finally, the user has the option to export the β-colour coded image in .tiff (which can be opened in Fiji for further analysis), image metadata and the parameters used for masking in .json format.

### Analysis of phase-separated vesicles

**Fig. 4.**
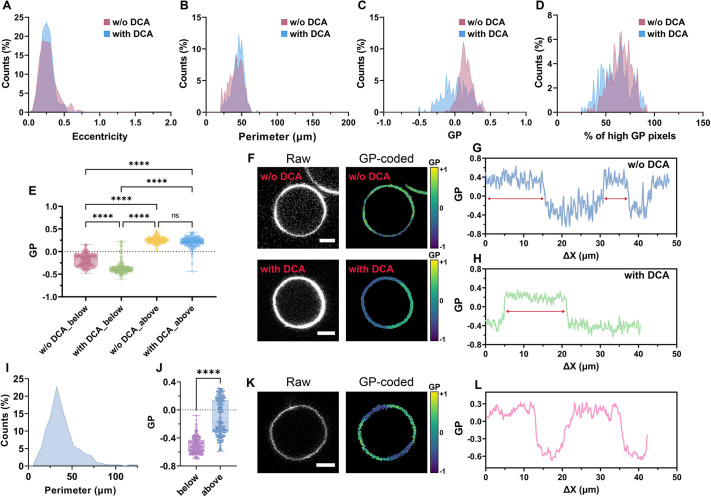
**VISION reveals differences in phase stability from synthetic vesicles.** (A–D) Distribution of vesicle eccentricity (A), perimeter (B), GP (C) and percentage of high-GP pixels (D) from phase-separated GPMVs with and without the addition of DCA. (E) Box plots reporting the median GP values of the two subsets of profiled points (falling either above or below the median GP from the profiled trajectory) from individual phase-separated GPMVs (*n*>100) without or with the addition of DCA from three independent biological replicates. ****P<0.0001; ns, not significant (assessed via non-parametric one-way ANOVA using the Kruskal–Wallis test). (F–H) Raw and GP colour-coded representative images (F) and profiled membrane (G,H) of a phase-separated GPMV with and without the addition of DCA. The red arrows highlight differences in phase lengths between the two conditions. (I,J) Distribution for the vesicle perimeter (I) and box plot (J) reporting the median GP values of the two subsets of profiled points (falling either above or below the median GP from the profiled trajectory) from individual phase-separated GUVs (*n*>220) from three independent biological replicates. ****P<0.0001 between the two subsets (non-parametric *t*-test analysis via the Mann–Whitney method). (K,L) Raw and GP colour-coded representative image (K) and profiled membrane (L) of a phase-separated GUV. Scale bars: 5 µm. In the profiled trajectories G, H and L, ΔX represents the lateral displacement along the vesicle's membrane in µm. Images of phase-separated GUVs reused from Ragaller et al. (2024) where they were published under a CC-BY 4.0 license. For box plots, the box represents the 25–75th percentiles, and the median is indicated. The whiskers show the range.

Thanks to the modules for single object detection and membrane profiling, VISION is very useful when characterizing phase-separated vesicles. In Fig. 4, we present an example of application for analysing phase stability in cell-derived giant plasma membrane vesicles (GPMVs). GPMVs differ from synthetic vesicles, such as GUVs, in that they retain majority of the membrane complexity of their cell precursors (Sezgin, 2022). Thus, we stained GPMVs with Pro12A (which reports on membrane fluidity) and investigated the effect of the chemical deoxycholic acid (DCA) on ordered-disordered phase transition in GPMVs by measuring changes in membrane fluidity (i.e. β-value) and phase separation efficiency. DCA is a bile acid naturally found in the human body. Its main function is emulsification and solubilization of fats in the body and it has been used as a biological modulating agent in immunology, cancer and cosmetics. It has been shown to stabilize phases with lower membrane order and to predominantly affect the disordered phase fluidity (Zhou et al., 2013). As shown in Fig. 4A,B, the addition of DCA had no visible effect on the vesicle morphology, which retained the same rounded shape and size distribution. On the other hand, a shift towards negative values was observed for the generalized polarization (GP; see section ‘Confocal and STED imaging’ in the Materials and Methods and Eqn S2 in the Fig. S1 legend for the equation for the GP calculation) distribution, suggesting that DCA changes the overall membrane fluidity in GPMVs. However, from the histogram of object-specific median GPs (Fig. 4C) it is not possible to conclude whether the observed shift reflects an increase in the total area of disordered domains per vesicle or an increase in fluidity of the existing domains. To this regard, by profiling the vesicle membrane along the equatorial plane, we were able to trace the spatial variation of GP from individual GPMVs and group the GP values measured at each point along the membrane into two distinct sets either above or below a defined threshold (i.e. the median GP value). Thus, we could observe a slight increase in the number of pixels (i.e. membrane areas) with lower membrane fluidity (Fig. 4D) in GPMVs treated with DCA. This evidence supports the hypothesis of a relative increase in the total surface area of low-GP phases upon DCA treatment. Furthermore, by plotting values from the two GP sets with and without the bile acid (Fig. 4E), we were able to confirm the results in literature (Zhou et al., 2013), which showed that DCA has no effect on the fluidity of ordered phases, but it further decreases the fluidity of the disordered areas. Besides yielding information on the overall extent and GP magnitude of the different phases, profiling via VISION can also provide insights into the morphology and size of domains in vesicles (Fig. 4F–H). Indeed, by looking at the profiled trajectories, it is possible to derive parameters such as the length of phases, the intra-phase heterogeneity of fluidity (i.e. fluctuations in GP values) and the gradient of phase separation (from the slopes of GP trajectory). To this regard, we found that the addition of DCA mainly leads to the formation of only two large phases with distinct GPs, instead of multiple smaller ones (Fig. 4G,H). For comparison, we also produced GUVs that spontaneously phase separate without the need of additional stabilizers. These synthetic vesicles showed similar morphology to GPMVs (Fig. 4I) but a different efficiency in phase separation. Indeed, as inferable from Fig. 4J, only half of the vesicles showed multiple phases with different membrane fluidity and lengths similar to the case of GPMVs with DCA (Fig. 4K,L).

### Correlation of membrane fluidity with mitochondrial potential

**Fig. 5.**
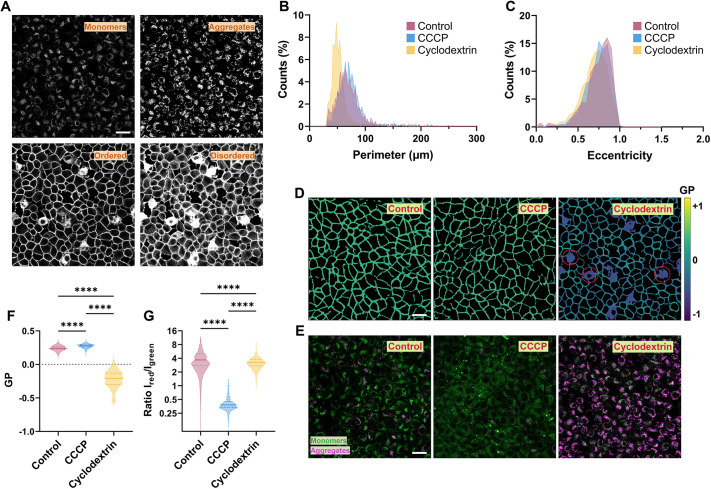
**VISION allows for correlation of membrane fluidity with mitochondrial potential.** (A) Four different intensity channels used to correlate fluidity of the plasma membrane with mitochondria membrane potential. ‘Ordered’ and ‘disordered’ refer to the channels used for the calculation of GP. (B) Distributions of cell perimeters for control and drug-treated samples. (C) Distribution of cell eccentricities for control and drug-treated samples. (D) GP colour-coded images from the three different samples. The colour bar represents the range of GP values. Red circles mark example cells with dye internalization. (E) Images showing the overlap between JC-1 monomers (green) and aggregates (magenta) from the three different conditions. (F,G) Violin plots showing the difference in GP (F) and ratio *I*_red_/*I*_green_ (i.e. mitochondrial potential, G) between the different conditions from three independent biological replicates (>1000 cells analysed). ****P<0.0001 (one-way ANOVA analysis using the non-parametric Kruskal–Wallis test). Scale bars: 25 µm. For violin plots, the 25–75th percentiles and the median are indicated.

VISION allows for simultaneous and independent characterization of multiple biophysical properties from both the plasma membrane and the cytosol. Thus, we utilized our software to investigate the correlation between remodelling of membrane fluidity (i.e. GP) and mitochondrial potential under stress. Specifically, we conducted experiments on epithelial-like rat kidney cells (NRK-52E) treated with either β-cyclodextrin or the protonophoric uncoupler carbonyl cyanide m-chlorophenyl hydrazone (CCCP). Cyclodextrin removes cholesterol from the plasma membrane (Zidovetzki and Levitan, 2007), whereas CCCP disrupts the potential of mitochondrial membrane by promoting the opening of the mitochondrial permeability transition pore (Ganote and Armstrong, 2003). As shown in Fig. 5A, we co-stained cells with Pro12A (for membrane fluidity) and JC-1 (for mitochondrial membrane potential; Sivandzade et al., 2019) and acquired the intensity from four different channels. The green and red channels were used to detect signal from JC-1 monomers and aggregates (used to mask mitochondria), respectively, and to calculate the mitochondrial membrane potential as the intensity ratio *I*_red_*/I*_green_. By contrast, the blue and cyan channels were used to detect signal from ordered and disordered phases, respectively, and to calculate the GP. Channel range was optimized to avoid spectral spillover of JC-1 into the Pro12A channels (Fig. S3). By performing single-cell characterization, we observed changes in cell morphology due to drug treatment. Specifically, both CCCP and cyclodextrin induced a slight decrease in cell perimeter (Fig. 5B) and eccentricity (i.e. more rounded cells, Fig. 5C), which could be ascribed to a partial cell detachment from the bottom of the well. Significant changes between control experiments and treated cells were also observed in membrane fluidity and mitochondrial potential (Fig. 5D–G). Whereas CCCP caused a slight decrease in membrane fluidity (i.e. higher GP values), the removal of cholesterol via cyclodextrin drastically lowered the median GP values (Fig. 5D,F), in agreement with data in literature (Fernández-Pérez et al., 2018). Treatment with the cyclodextrin also promoted dye internalization (red circles in Fig. 5D) as a consequence of the disruption of lipid bilayer packing (Fig. 5D). Interestingly, the two drugs also had an opposite effect on the mitochondria membrane potential. CCCP strongly impaired mitochondria health, as shown from an overall decrease in JC-1 aggregates (Fig. 5E, green versus magenta) and a lower *I*_red_/*I*_green_ ratio from masked mitochondria (Fig. 5G), as reported in the literature (Miyazono et al., 2018). By contrast, cyclodextrin seemed to slightly increase both the number of JC-1 aggregates per cell (Fig. 5E, magenta) and the mitochondrial potential (Fig. 5G). This hyperpolarization of mitochondria could be a direct consequence of the cholesterol depletion from the plasma membrane which, in the short term, would trigger recruitment of cholesterol from membranes from other organelles to counteract the effect.

### Protein accumulation at the contact site between two cells

**Fig. 6.**
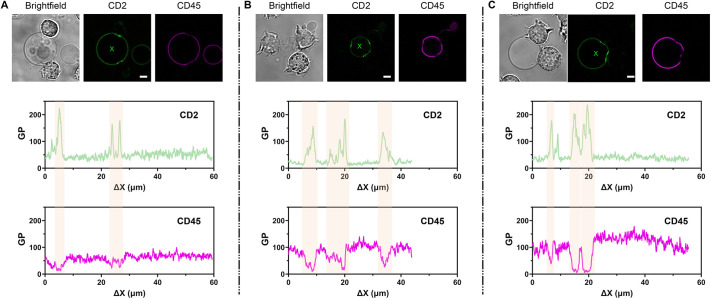
**VISION reveals protein cluster formations at the cell-to-GUV contact site.** (A–C) Examples of Jurkat T cells interacting with GUVs decorated with CD2 and CD45 adhesion proteins. The green and magenta channels refer to CD2 and CD45 protein, respectively. The pink areas in the profiled trajectories highlight either accumulation or exclusion of proteins from the membrane-membrane contact area. We analysed contact areas from three different biological replicates (>45 GUVs). Scale bar: 5 µm. In the profiled trajectories, ΔX represents the lateral displacement along the vesicle membrane in µm.

The applications of VISION are not limited to the study of biophysical parameters, as our software can be used to study any multi-parametric aspect of images. For this purpose, we also tested the software applicability in studying cell–cell contact areas, which are crucial for intercellular communication, cell polarization and tissue integrity (Garcia et al., 2018). Specifically, we focused on the redistribution of fluorescently labelled signalling proteins at a reconstituted immune synapse (IS) by measuring the respective fluorescence intensities (i.e. β-value). The IS describes a highly spatio-temporally organized contact between immune cells and their target antigen-presenting cells resulting in the activation of immune signalling (Dustin, 2014; Monks et al., 1998). The spatial organization of proteins within the IS resembles a bull's eye-like structure comprising supramolecular activation clusters (SMACs), which are characterized by focal accumulation or exclusion of certain proteins (Dustin, 2014). The enrichment of the two adhesion receptors CD2 and CD58 at the cell–cell contact is important for IS formation and function (Dustin, 2014; Sanchez-Madrid et al., 1982; Shaw et al., 1986). By contrast, the exclusion of the tyrosine phosphatase CD45 from the IS ensures proper regulation of the immune signalling process (Dustin, 2014; Chang et al., 2016). In Fig. 6, we show the results obtained from a minimal system resembling the IS, which consisted of GUVs decorated with CD2 and CD45 (1:1 ratio) in contact with Jurkat T cells. As expected, we observed the enrichment of CD2 and a corresponding exclusion of CD45 at the cell–GUV contact, thus confirming previous observations on similar systems (Céspedes et al., 2021; Jenkins et al., 2018). Interestingly, from the analysis of the two intensity trajectories – green for CD2 and magenta for CD45 – we observed a quite broad heterogeneity in protein accumulations and/or exclusion (i.e. signal intensity) from the contact area and the tendency of CD2 to form focal clusters within the same IS (Fig. 6). Therefore, our software can be used to profile cell–cell contact sites at a submicrometre spatial resolution and study the biological significance of protein clustering or redistribution within those areas in various scenarios.

### Profiling nanodomains with super-resolution

**Fig. 7.**
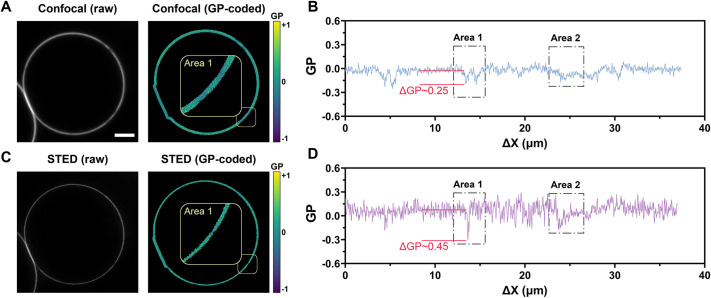
**VISION can profile membrane fluidity with super resolution.** (A,B) An example of a phase-separated GUV stained with Di-4-AN(F)EPPTEA imaged with confocal microscopy (A) and its corresponding profiled trajectory (B). (C,D) An example of phase-separated GUV imaged with STED microscopy (C) and its corresponding profiled trajectory (D). Scale bar: 3 µm distance. Images of GUVs reused from Sezgin et al. (2017) where they were published under a CC-BY 4.0 license.

As mentioned above (Fig. 3), VISION adopts a different approach to profile the lipid bilayer of vesicles by sliding a customizable moving average filter along a membrane-centred guideline. By doing so, it ensures a constant area of integration which is independent from the local membrane morphology. This is crucial to obtain quantitative measurements of small variations in the membrane biophysical properties under investigation. Furthermore, since the size of the integrating element ultimately depends on the image pixel size (with the smallest element being a 3×1 pixels line), by operating at high spatial resolution it is possible to capture heterogeneities occurring at the nanoscale. This is shown in Fig. 7A, where we compare the GP profiles measured with either confocal or stimulated emission depletion (STED) microscopy from the same phase-separated GPMV. In both scenarios, the pixel size was set to ∼32 nm and a 3×3 pixel averaging filter was chosen for GP integration, thus yielding a total area of ∼0.08 nm^2^ per profiled point (i.e. spatial displacement ΔX). From the two areas highlighted in Fig. 7B–D, it appears clear how both the spatial resolution and contrast in GP magnitude (i.e. ΔGP) between phases is enhanced in STED versus confocal. Thus, our software can be used to investigate the dynamic of biophysical remodelling of membranes at both the micrometre and nanometre scale.

## DISCUSSION

With the advancement in technologies, microscopy is quickly overcoming the historical limitation in throughput and dimensionality. New instrumentation offers the possibility of extending the collectable information beyond the spatial and temporal space to the spectral dimension, thus delivering improved multiplexing (Huang et al., 2022). VISION was developed to take advantage of the multi-channels and spectral modalities implemented in many commercial microscopes and perform complex mathematical operations from up to four different channels. Thanks to the different modules implemented, our software finds large applicability in a plethora of scenarios spanning from correlative analysis between the plasma membrane and the cytosol or subcellular compartments to experiments on protein colocalization and to profiling cellular properties with ratiometric environment-sensitive probes. Furthermore, the high versatility of the module for image masking allows to work with a variety of biological samples, such as vesicles (e.g. GUVs, GPMVs), live cells or more-complex tissue images. In addition, the novel approach VISION uses for membrane profiling enhances the accuracy of signal integration, potentially improving quantitative analysis of, for example, protein clustering or accumulation at cell–cell junctions (Shashikanth et al., 2016), spatial-temporal remodelling of membrane biophysical properties and the dynamics of phase separation.

### Future developments

Given the increasing interest in studying biophysical properties of cell and organelle membranes, we intend to extend the capability of our software by introducing extra functionalities to perform adaptive thresholding – which is useful when working with *z*-stack and *t*-stack of images with heterogenous brightness – and AI-based segmentation. Currently, segmentation of the image occurs at the object level, to identify individual cells from clusters. In the future, we plan to implement an additional module for intra-cytosol particle detection, which will further broaden the applicability of our software to studies on subcellular structures. Finally, we will introduce the possibility for performing single-particle or single-cell tracking analysis of temporally resolved images, and to perform spectral deconvolution of the emission spectrum, thus increasing the multiplexing capability.

## MATERIALS AND METHODS

### Licenses, GUI development and standalone executables

VISION software, the python core-code, user guide, troubleshooting and tutorials can be freely downloaded from the GitHub repository: https://github.com/biosciflo/VISION. Video tutorials can be found at: https://www.youtube.com/watch?v=ZDZju8mgNiY and https://www.youtube.com/watch?v=NmAVgJb-E-g; For the GUI development and distribution of standalone executables, the VISION software is licensed under GPL-3.0. The standalone software application VISION features a graphical user interface (GUI) implemented using Python and the Qt framework (PyQt). The GUI was designed to facilitate user interaction and streamline analysis tasks, including image loading, preprocessing, analysis algorithms and result visualization. The software was packaged using PyInstaller to create standalone executables for Windows and macOS platforms, enabling easy deployment and usage across various operating systems. This approach ensures accessibility and usability for researchers in the field of microscopy image analysis, offering a user-friendly solution for their analytical needs.

### Preparation of synthetic vesicles

To generate synthetic vesicles different lipids were mixed at different ratios, namely POPC (Avanti Polar Lipids, cat# 850457C), DOPC (Avanti Polar Lipids, cat# 850375C), egg sphingomyelin (SM, Avanti Polar Lipids, cat# 860061C), cholesterol (Avanti Polar Lipids, cat# 700000P) and DGS-NTA(Ni) (18:1, Avanti Polar Lipids, cat# 790404C). For normal GUVs, POPC was mixed with 2% DGS-NTA(Ni), whereas phase-separated GUVs were obtained from a SM:DOPC:cholesterol lipid mixture at 2:2:1. Then, vesicles were generated by electroformation using a custom-built GUV Teflon chambers with two platinum electrodes (Angelova and Dimitrov, 1986; Sezgin et al., 2012b) according to a previous protocol (Sezgin et al., 2012a). A volume of 6 μl of 1 mg/ml lipid in chloroform was homogeneously distributed on the electrodes and dried under a nitrogen stream. After placing the electrodes in 370 μl of 300 nM sucrose solution (370 μl), electroformation was performed at 2 V and 10 Hz for 1 h followed by 2 V and 2 Hz for 30 min. Phase-separated GUVs were prepared above the specific lipid transition temperature at 70°C, whereas the other GUVs were generated at room temperature (RT). GPMVs were generated from human epithelial HeLa cells following a slightly modified version of a protocol previously described (Sezgin et al., 2012a) In brief, cells were washed twice with HBSS (Thermo Fisher Scientific, cat# 15266355) and incubated for 3 h at 37°C and 5% CO_2_ with GPMV-vesiculation buffer consisting of 25 mM methanol-free formaldehyde (Thermo Fisher Scientific, cat# 11586711) and 2 mM dithiothreitol (Bio-Rad, cat# 1610610) dissolved in HBSS. After formation, one batch of GPMVs was incubated with 0.6 mM of deoxycholic acid (Sigma-Aldrich, cat# D2510) as described previously (Zhou et al., 2013) to stabilize phase separation at RT.

### Cells maintenance

Commercial cell lines were purchased from the ATTC [HL-60 (cat # CCL-240), NRK-52E (cat# CRL-1571), Jurkat (clone E6-1, cat# TIB-152), HeLa (cat# CRM-CCL-2)].

Cells were cultured in either RPMI with L-glutamine (Thermo Fisher Scientific, cat# 12004997), for suspension cells and HeLa cells, or DMEM with high glucose (Thermo Fisher Scientific, cat# 11965092) supplemented with 10% fetal bovine serum (Sigma-Aldrich, cat# F7524) at 37°C and 5% CO_2_, for adherent cells. Before experiments on cell–GUV contacts, cells were washed twice in PBS at 1500 rpm for 1 min at RT and subsequently counted. For imaging, ∼3×10^4^ cells per well were added to each well containing GUVs in μ-Slides (18-well glass bottom, ibidi, cat# 81817). For GPMV preparation, cells were seeded into 12-well plates to reach a confluency of 70–80% on the day of experiment and transferred into an eight-well chambered cover glass (Cellvis, cat# C8-1.5H-N) precoated with fetal bovine serum albumin (Sigma-Aldrich, cat# A3803). For drug re-treatment experiments, NRK cells were seeded into eight-well polymer bottom Ibidi slides at 70% confluency (#1.5, cat# 80801) a day before the analysis.

### Protein labelling of GUVs

The proteins CD2 (Human, Recombinant, ECD, His Tag from SinoBiological, cat# 10982-H08H) and CD45 (Human, Recombinant, ECD, His Tag from SinoBiological, cat# 16884-H08H) were labelled with Alexa Fluor™ 488 NHS Ester (Succinimidyl Ester, Thermo Fisher Scientific, cat# A20000) (AF488) and Alexa Fluor™ 647 NHS Ester (Succinimidyl Ester, Thermo Fisher Scientific, cat# A37573) (AF647), respectively. The amount of NHS Ester dye needed for the labelling reaction was calculated following Eqn 1:
(1)

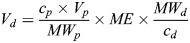
where *V_d_* is the volume of the dye solution, *c_p_* is the protein concentration, *V_p_* is the volume of protein, *MW_p_* is the protein molecular mass, *ME* is the molar excess (number of dye molecules per protein molecule, here we chose 5) and *c_d_* is the concentration of the dye solution. To each 1 ml protein solution, 0.1 ml NaHCO_3_ (1 M) solution in water pH 8.5 was added. The calculated amount of dye solution (in DMSO) was added to the protein solution and incubated at RT for 1 h with continuous stirring. The protein–dye solution was then added to two consecutive 0.5 ml 40K MWCO Zeba™ Spin Desalting Columns (Thermo Fisher Scientific, cat# 87767) (previously washed twice with PBS) and centrifuged at 1500 ***g*** for 2 min.

### Drug treatments

For experiments on cholesterol depletion, a 10 mM solution was prepared by dissolving 0.12 g of methyl-β-cyclodextrin in 10 ml L-15 medium (Sezgin et al., 2015). NRK-52E cells were then incubated for 30 min at 37°C and 5% CO_2_ in 300 µl/well of cyclodextrin solution together with JC-1. For experiments on disruption of the mitochondrial membrane potential, CCCP was added to cells at a final concentration of 20 µM and incubated at 37°C and 5% CO_2_ for the last 5 min of incubation with JC-1 (Sivandzade et al., 2019). After treatments, cells were washed twice with PBS, and 300 µl/well of fresh L-15 was added before imaging.

### Labelling of membranes and mitochondria

All fluorescent dyes used for plasma and mitochondrial membrane labelling, namely JC-1 (Thermo Fisher Scientific, cat# T3168), NR12S (Biotechne, cat# 7509), NR12A and Pro12A (received from our collaborator Prof. A. S. Klymchenko, UMR 7021 CNRS, Université de Strasbourg, France) were dissolved in DMSO to make a stock solution of given concentration. Stock solutions were used to stain phase-separated GUVs at a final concentration of 100 nM of NR12S or NR12A and 300 nM Pro12A, and stain phase-separated GPMVs with 400 nM of Pro12A. Images were acquired after ∼5 min from staining. For confocal imaging of cell–GUV contacts, the GUVs were stained with 0.05 µg of both CD2-488 and CD45-AF647 at RT for 30 min. NRK-52E cells were stained with JC-1 at a final concentration of 800 nM for 3×10^5^ cells/well in Leibovitz's L-15 medium (Thermo Fisher Scientific, cat# 21083027) and incubated for 30 min at 37°C and 5% CO_2_ (Sivandzade et al., 2019). Then, cells were washed twice with fresh L-15 buffer and stained with NR12A at a final concentration of 500 nM.

### Confocal and STED imaging

Confocal images were acquired using a Zeiss LSM 780 confocal microscope with a 32-channel array of gallium arsenide phosphide (GaAsP) detectors or a Zeiss LSM 980 confocal microscope AIRY2/FCS (for imaging of cell–GUV contact areas). For the LSM 780, the wavelengths range for each detector was set to 9 nm. Pro12A was excited using a 405 nm laser, whereas NR12A and NR12S were excited using a 488 nm laser. Different ranges of wavelengths were acquired for the different membrane-intercalating probes, namely ∼423–601 nm for Pro12A and ∼503–700 nm for NR12A and NR12S (Ragaller et al., 2022). Images were acquired using a C-Apochromat 40×/1.20 W Korr water immersion objective Depending on the experiment, we used the following settings: dichroic beam splitters MBS-405, MBS-488 or MBS-488/639, laser power ∼0.5–5 µW, detector gain ∼680–800, pixel dwelling time ∼2–3 µs and pixel size ∼100–200 nm. When acquiring spectral images, we recorded the full emission spectrum of the probe, selected two channels (each of them covering a 9-nm range of wavelengths) representative of ordered and disordered phases, and used them to calculate the corresponding GP value, according to Eqn S2. By recording the full emission spectrum, we were able to choose the best pair of channels for ordered and disordered signals, which ensured the highest resolution in GP. For experiments on cell–GUV contacts, CD2–AF488 was excited at 488 nm CD45–AF647 at 633 nm. For correlation experiments between membrane fluidity and mitochondrial membrane potential, we performed sequential excitation and emission acquisition. Specifically, JC-1 dye was excited with a 488 nm laser and the detection range was set either at 500–550 nm (for JC-1 monomers) or 580–610 (for JC-1 aggregates). Instead, Pro12A was excited with a 405 nm laser and the detection range was set either at 420–510 nm (for ordered) or 470–510 (for disordered).

STED images were acquired using a Leica SP8 STED microscope (Leica Microsystems, Mannheim, Germany) equipped with a HC PL APO C S2 100×/1.40 oil objective, a pulsed (80 MHz) white light laser (WLL), and a pulsed 775 nm STED laser. For excitation, we selected 488 nm from the WLL. The master laser power for the WLL was 70 µW and for the STED laser ≈280 mW (maximum power, measured at the back aperture of the objective). The laser light was further filtered using Notch Filters (NF) 488/561/633 nm (for the excitation) and 775 nm (for STED). Before each measurement session, the beam alignment of the WLL and the STED laser was checked. The alignment was performed with the 592 nm STED laser of the microscope (reference laser). The emission was detected using hybrid detectors. When two wavelength ranges were needed, 520–570 nm and 620–700 nm intervals were selected. Detector gating of 1.5–6 ns was applied. Two image sequences were taken; the first sequence without and the second sequence with the STED laser on. The first sequence provided the standard confocal image whereas the second sequence the high-resolution STED microscopy image. The scanning speed was set to 1800 Hz line frequency and a line-averaging over four lines was applied. The pixel size was optimized for the STED microscopy image and kept the same for confocal (≈20 nm/pixel).

### Statistical analysis

Statistical analysis on phase-separated GPMVs and GUVs was done in Prism GraphPhad Version 9.4.1. Statistical significance between the different subsets was assessed via either non-parametric Mann–Whitney *t*-test (for GUVs) or non-parametric one-way ANOVA using the Kruskal–Wallis test (for GPMVs).

## Supplementary Material



10.1242/joces.262166_sup1 Supplementary information
