# Supplementary material for: VISION – an open-source software for automated multi-dimensional image analysis of cellular biophysics
Source: J Cell Sci. 2024 Oct 23;137(20):jcs262166. doi: 10.1242/jcs.262166 (PMC11529879; doi:10.1242/jcs.262166)
Supplement: Supplementary information [file joces-137-262166-s1.pdf]

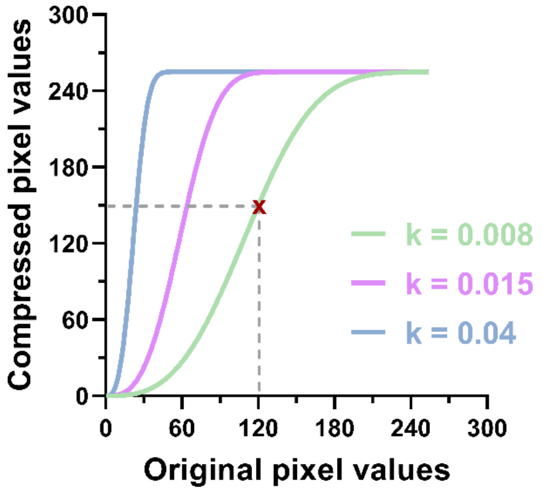

Fig. S1. Function for compression of pixel's values. By increasing the value of *k* parameter, pixels are compressed towards higher values.

In Fig. S1, we show the effect of enabling the function for compression of pixel values during the masking. The function is described in Eq. S1:

$$I_n = I_{max} - I_{max} * e^{(-k^3 * I_o^3)} \qquad \text{Eq. S1}$$

I_{max}, I_n and I_o refer to the maximum (*e.g.*, 255 for an 8-bit image), the compressed and the original pixel value, respectively.

Equation for the Generalized Polarization (GP)

GP was calculated according to literature (Parasassi *et al.*, 1990), following the equation:

$$GP = \frac{I_o - I_d}{I_o + I_d} \qquad \text{Eq. S2}$$

where Io and Id refer to the emission intensity measured from the ordered and disordered membrane phase, respectively.

Journal of Cell Science • Supplementary information

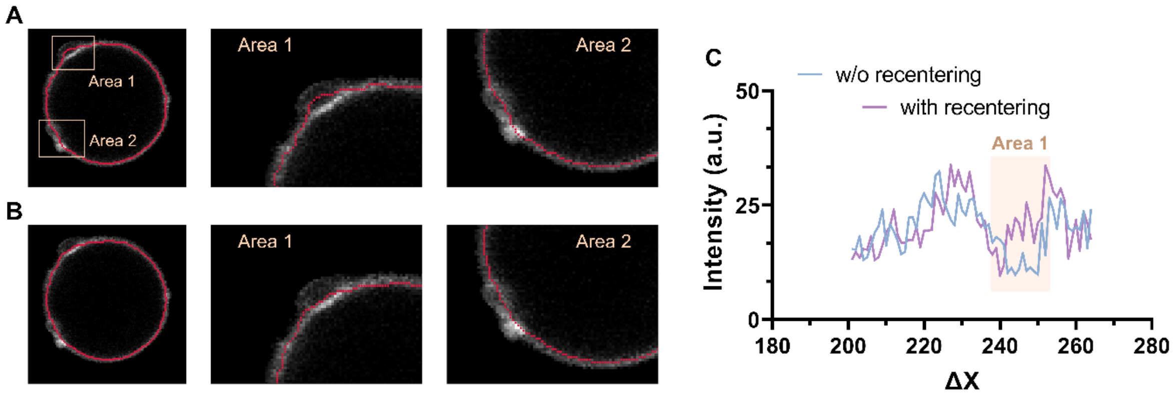

Fig. S2. Recentering of the membrane guideline for profiling. (A-B) Comparison between guidelines for membrane profiling obtained either without (A) or with (B) recentering the guideline. (C) Profiled intensities from one of the channel used to calculate the β-value. As visible from the figure inserts, the recentering option is useful in the presence of ‚blobs' to ensure proper profiling of the membrane. If the line is already centred onto the membrane, then, the recentring function has little to no effect (area 1 *vs* area 2).

Journal of Cell Science • Supplementary information

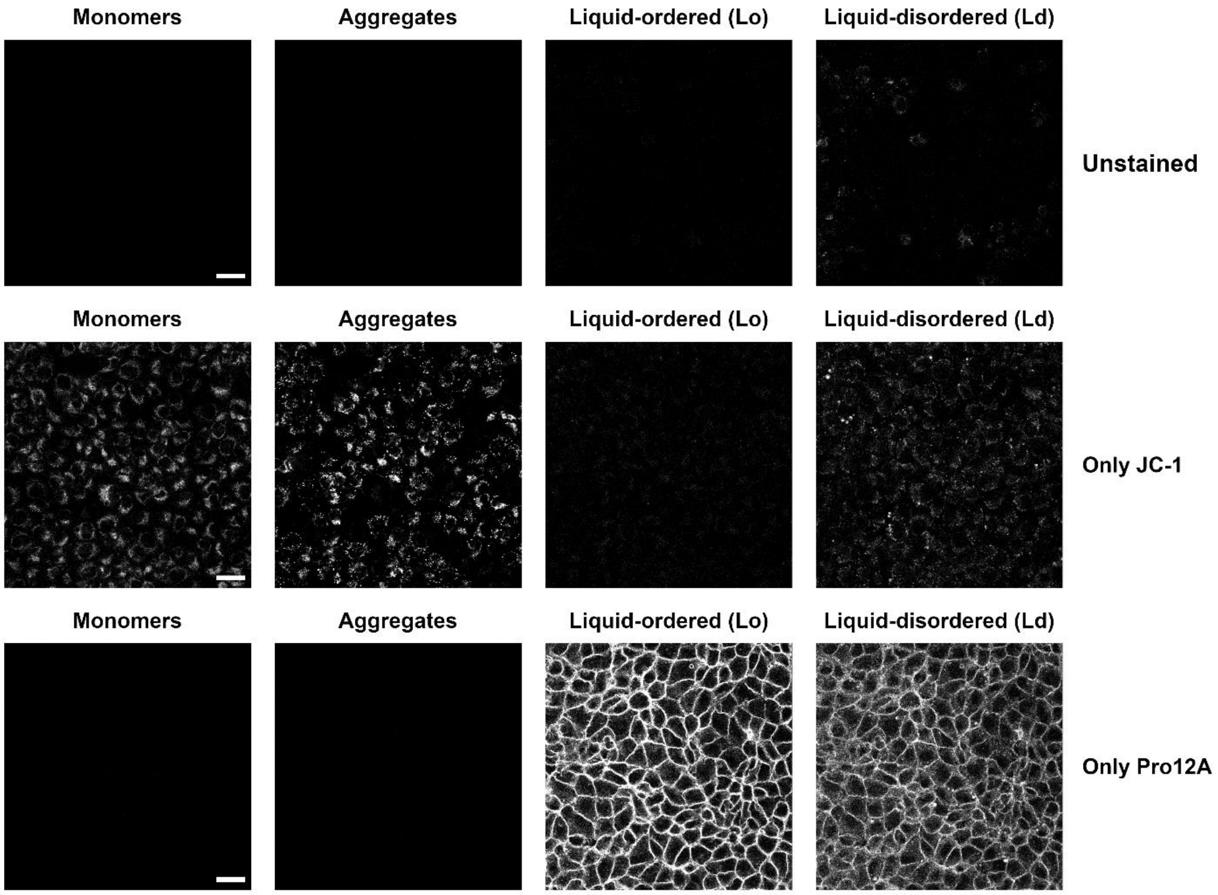

Fig. S3. Reciprocal spectral spillover of JC-1 and Pro12A. Rows show different experimental conditions wherease columns refer to the specific channel used to measure the intensity from the JC-1 monomers, JC-1 aggregates, the membrane liquid-ordered and liquid-disordered phases. Images were acquired using the same settings: exitation at 405 nm, laser power = 1 mV (4% of 25mV), detectors gain = 730 and wavelengts range = 420 –450 nm (Lo) and 470 – 510 nm (Ld) for Pro12A and exitation at 488 nm, laser power = 0,09 mV (0,3% of 30mV),detectors gain = 680 and wavelengts range = 500 – 550 nm (monomers) and 580 – 610 nm (aggregates) for JC-1.As shown in the figure, Pro12A does not spill over the channels used to measure the mitochondria membranepotential. Similarly, JC-1 has negligible (~1/15 of Pro12A signal) spillover only into the channel for Ld. However, the masking for membrane fluidity is performed on the Lo channel, thus avoiding interference from JC-1. The scale bars indicate a 25 μm distance.

Journal of Cell Science • Supplementary information

Graphical User Interface

In Figures S4-S7 we showcase the interface of our software VISION. Coloured boxes in Figure S4 highlight the main sections in the interface: a section for images uploading and selection (red box); four different tabs (yellow box) for setting the parameters for masking and analysis of both the membrane and cytosol, as well as for data saving; three different tabs (blue box) for visualization of the results and a console window (pink box) where the users can read potential errors messages during the analysis.

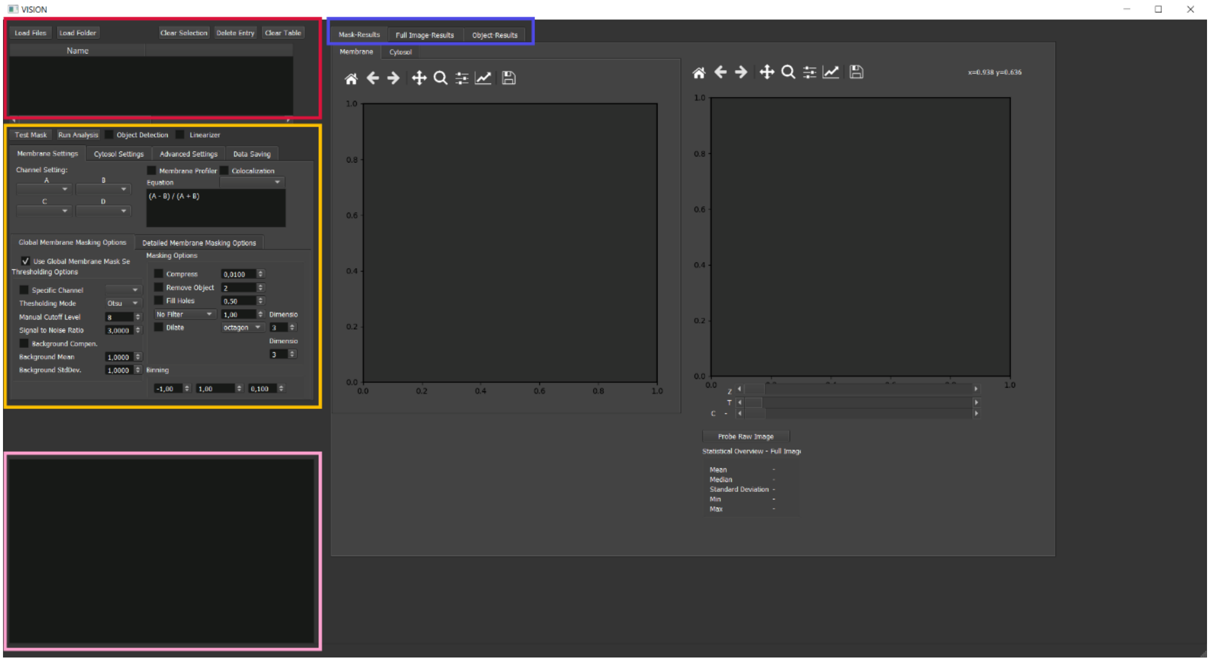

Fig. S4. Showcase image of the main sections of the GUI for the software VISION.

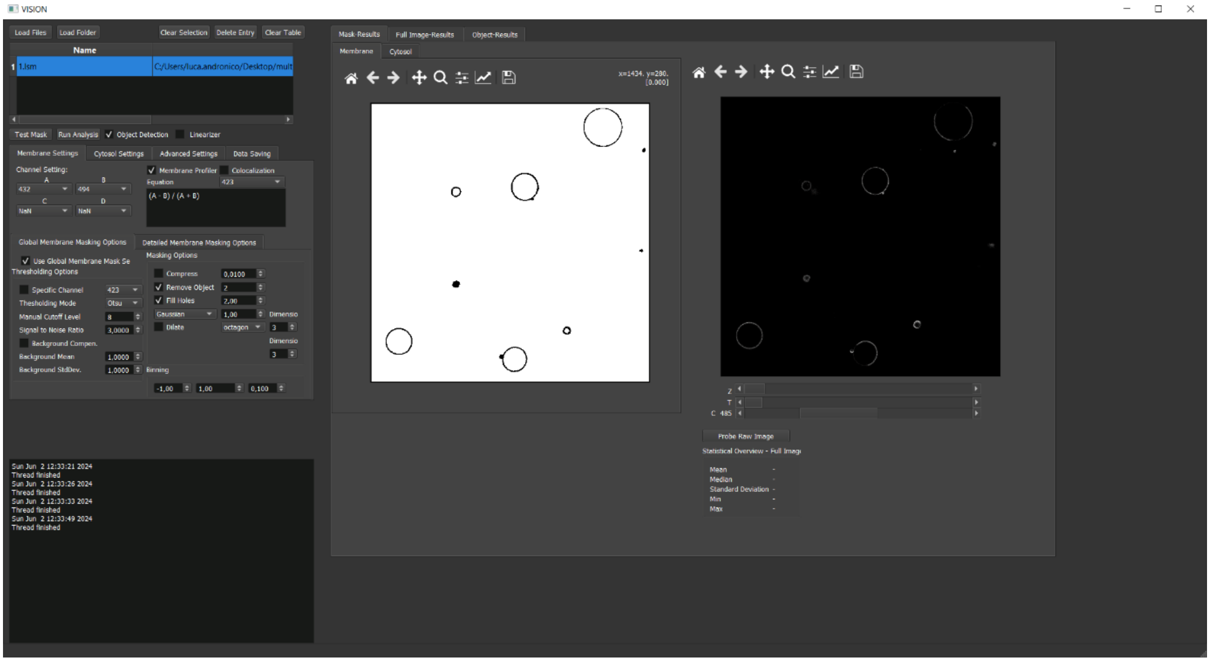

Fig. S5. Showcase image showing the masking results for the selected image. The left and right images show the mask and the raw image, respectively.

Journal of Cell Science • Supplementary information

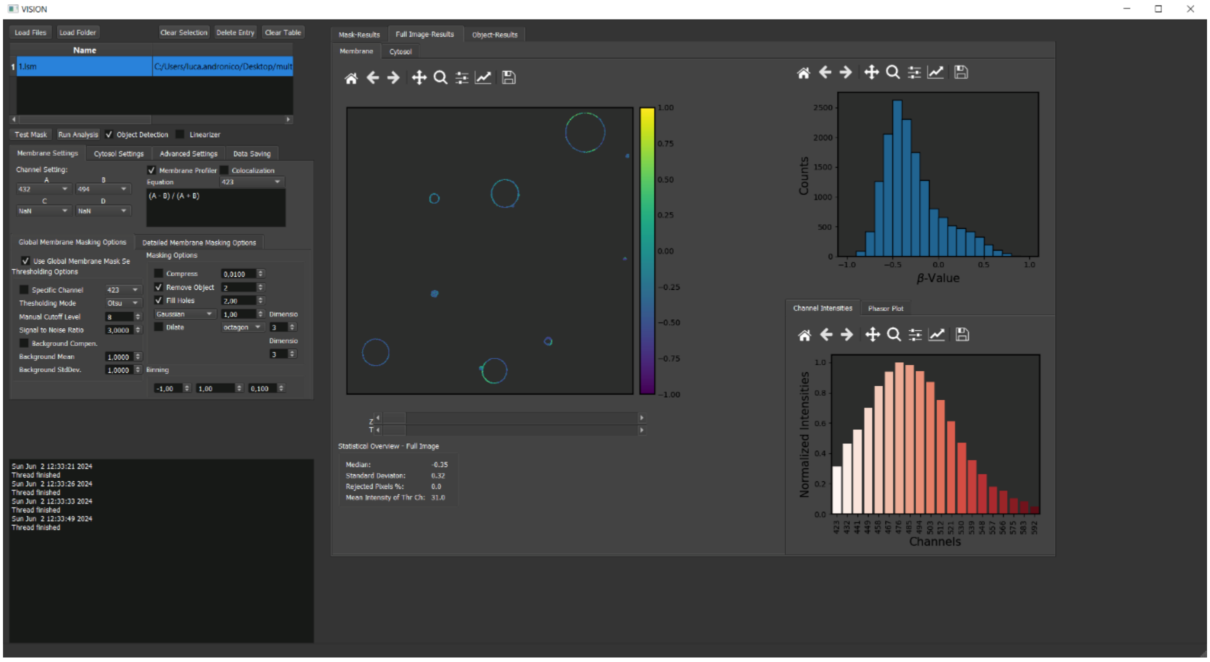

Fig. S6. Showcase image showing the „full image“ results from the selected image. The left window shows the β-colour coded image; the top-right plot (blue-coloured) shows the histogram of pixel-wise β values from the whole picture and the top-bottom plot (red-coloured) shows the emission spectrum (*i.e.*, the emission intensity from individual channels) of the dye used to measure the membrane’s biopysical properties.

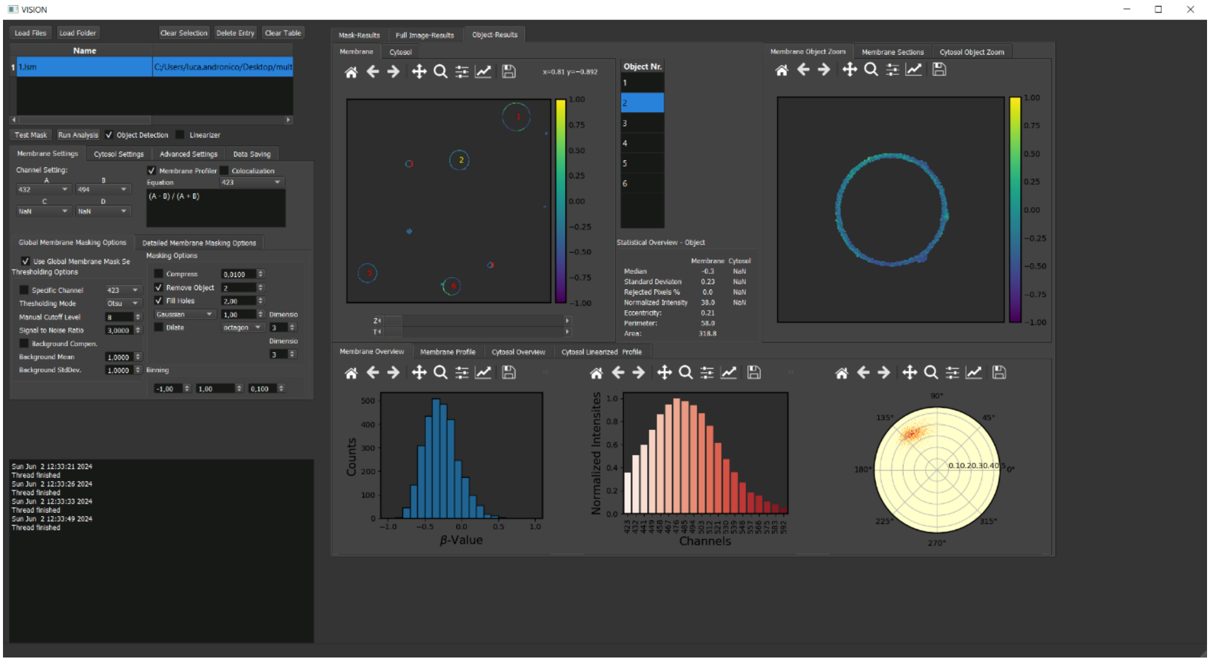

Fig. S7. Showcase image showing the single-object results from the selected image. The left window shows the β-colour coded image with identified objects which can be individually selected usiung the numbered list; the top-right window shows the β-colour coded image of the selected object; the bottom plots (from left to right) show the histogram of β values, the emission spectrum and the phasor plot of the selected object, respectively.

References

Parasassi, T. *et al.* (1990) ‘Phase fluctuation in phospholipid membranes revealed by Laurdan fluorescence.’, *Biophysical Journal*, 57(6), pp. 1179–1186.
