# Supplementary material for: VISION – an open-source software for automated multi-dimensional image analysis of cellular biophysics
Source: J Cell Sci. 2024 Oct 23;137(20):jcs262166. doi: 10.1242/jcs.262166 (PMC11529879; doi:10.1242/jcs.262166)
Supplement: Supplementary file 1 [file joces-137-262166_review_history.pdf]

VISION - an open-source software for automated multi-dimensional image analysis of cellular biophysics

Florian Weber, Sofiia Iskrak, Franziska Ragaller, Jan Schlegel, Birgit Plochberger, Erdinc Sezgin and Luca A. Andronico
DOI: 10.1242/jcs.262166

Editor: Guillaume Jacquemet

Review timeline

Original submission:	29 March 2024
Editorial decision:	9 May 2024
First revision received:	25 July 2024
Accepted:	2 September 2024

Original submission

First decision letter

MS ID#: JOCES/2024/262166

MS TITLE: VISION - an open-source software for automated multi-dimensional image analysis of cellular biophysics

AUTHORS: Florian Weber, Sofiia Iskrak, Franziska Ragaller, Jan Schlegel, Birgit Plochberger, Erdinc Sezgin, and Luca Andronico
ARTICLE TYPE: Tools and Resources

We have now reached a decision on the above manuscript.

To see the reviewers' reports and a copy of this decision letter, please go to: https://submit-jcs.biologists.org and click on the 'Manuscripts with Decisions' queue in the Author Area. (Corresponding author only has access to reviews.)

As you will see, the reviewers raise a criticisms that prevent me from accepting the paper at this stage. They suggest, however, that a revised version might prove acceptable, if you can address their concerns. If you think that you can deal satisfactorily with the criticisms on revision, I would be pleased to see a revised manuscript. We would then return it to the reviewers.

Please ensure that you clearly highlight all changes made in the revised manuscript. Please avoid using 'Tracked changes' in Word files as these are lost in PDF conversion.

I should be grateful if you would also provide a point-by-point response detailing how you have dealt with the points raised by the reviewers in the 'Response to Reviewers' box. Please attend to all of the reviewers' comments. If you do not agree with any of their criticisms or suggestions please explain clearly why this is so.

Reviewer 1

Advance summary and potential significance to field

© 2024. Published by The Company of Biologists under the terms of the Creative Commons Attribution License (https://creativecommons.org/licenses/by/4.0/).

In the manuscript by Weber, F. and colleagues, the authors present an open-source software package, VISION, for automated segmentation and analysis of multidimensional images. They then go on to demonstrate its application for assessment of probes for membrane fluidity, mitochondrial healthy and individual protein localisation, confirming several findings from previous studies, while also expanding on these findings through the multidimensional analysis. Overall, the study is well presented, and the software is likely to provide a powerful tool to microscopists, particularly with the automated segmentation that they have implemented within the platform. However, some additional controls and details should still be added in order to support the interpretation of the experimental data. Please consider the following questions and comments.

Comments for the author

Major points:

1. It is unclear in Figs. 4-7 the number of biological replicates that were performed to accumulate the n values presented. Please indicate the biological and technical replicates clearly in all figure legends.

2. It is not clear that “lateral displacement” is “ΔX”. This is in several figures, but only explained in the legend for Fig. 3. Could you explain this in all legends? Also, why is this in pixel units and not in nanometres, for example? That would help to get an idea as to the effect of vesicle size on the distance between membrane regions, or is this necessary to normalise data between different vesicle types? Is this somehow different in Fig. 7 where an approximate area was calculated from this value?

3. Given your conclusion in the “Analysis of phase separated vesicles” section (page 8) is that the DCA had no effect on ordered phases, but further increased the fluidity of the ordered phases, could you compare the binned values from the “GP_below” and “GP_above” in Fig. 4E & 4F? As I understand it, there should be a significant difference between the “GP_below” values in 4E & 4F, but not between the “GP_above”, as they should correspond roughly to the disordered and ordered membrane regions respectively. Can you also further comment on the increase in disordered regions, as the reference you give (Zhou, et al., 2013, JBC) used a system where the membrane order could not change. Do you think that you are seeing an additional effect of DCA here in decreasing the fraction of ordered membrane regions?

4. Fig. S3 assessing the spectral spillover between the JC-1 and Pro12A probes is necessary, but performed in a way that doesn’t properly assess the spillover of either probe. To assess the bleed-through of Pro12A into the JC-1 channels, cells should be treated with Pro12A or JC-1 alone and assessed using the same excitation and emission channels as would be used for the co-stained cells. The bleed-through from this control experiment should then be presented in Fig. S3 to show the bleed-through of Pro12A into the JC-1 emission channels after excitation with the 405 and 488 nm laser lines. The same should be done in the JC-1 stained cells to assess bleed-through into the Pro12A channels from both 405 and 488 excitation.

Minor points:

1. “Python” is misspelled in the abstract and should be corrected from “Phyton”. There are a couple of other spelling mistakes and small grammatical errors in the manuscript that should also be corrected to enhance an otherwise clear and logical manuscript text.

2. The “(-)” and “(+)” are not very clear for the DCA treatments. Could you instead have “(-)DCA” and (+)DCA”?

3. The details of the statistical analysis given in the legend for Fig. 4 (and in the “Statistical analysis” section of the methods) are incomplete. Please indicate the specifics of the “non-parametric t-test performed”. For example, was it a Mann-Whitney or Kolmogorov-Smirnov test?

4. When referring to the lack of change of vesicle morphology after addition of DCA (page 8), the reference to Fig. 4A-C should be corrected to Fig. 4A & 4B. Fig. 4C refers to the GP and not the size/morphology, which you discuss in the next sentence.

© 2024. Published by The Company of Biologists under the terms of the Creative Commons Attribution License (https://creativecommons.org/licenses/by/4.0/).

5. The fluorescence spectrum of several dyes, including Pro12A, is modified based on the solvent used to solubilise the dye. Could you please provide the details for the solvents used and the suppliers for Pro12A, JC-1, NR12A and NR12S?

Reviewer 2

Advance summary and potential significance to field

The manuscript titled “VISION — an Open-Source Software for Automated Multi-Dimensional Image Analysis of Cellular Biophysics” by Florian Weber et al introduces a new open-source software for bioimage analysis of spectral imaging microscopy. The manuscript comprehensively describes all modules and features of the VISION software and illustrates them with various biophysical examples and applications. VISION is an open-source Python-based software released under the GPL-3.0 License. While the manuscript presents interesting findings and the potential value of the new software to the biophysical community, there are several concerns that should be addressed before publication. In the “Suggestions to Authors” section, I provide a list of these concerns in the hope of improving the final manuscript and the VISION software.

Comments for the author

1. At the outset of the manuscript, the authors missed an opportunity to credit the original work that proposed the GP function, opting instead for their own literature. This omission extends to other methods, like spectral phasor plots or GUV electroformation. I strongly recommend referencing the original works of Parasassi et al. (1991) for GP, Fereidouni (2012) for Spectral phasor plots, and Angelova 1986).

2. While the authors claim support for several file formats, only Zeiss formats (czi, lsm) or OME-tiff are guaranteed. I suggest tempering this claim or devising protocols to open files from Leica, Olympus, or Nikon.

3. Please provide a description and mathematical notation for the b-values calculated in the paper.
It wasn’t clear until late in the manuscript which b-values were used, and there was no description of the mathematics involved.

4. The use of spectral phasor plot representation and analysis on b-value histograms is confusing.
It seems like a backward use of a model-free method. First, a b-value is calculated with a mathematical model, and then the results are represented using a model-free approach. This is particularly puzzling for the GP, given the existing literature on spectral phasor analysis of LAURDAN fluorescence.

5. There is no mention of other software used in the field for spectroscopy on images. Some examples include SimFCS, Spectral image analysis (Fiji module), Spectral phasor Fiji plugin, and PhasorPy for phasor analysis of FLIM and HSI data. It’s advisable to acknowledge them.

6. What are the file formats for exporting the b-value images generated? This is not mentioned in the manuscript. Also, exporting data to an Excel spreadsheet in a proprietary format is not ideal for working in an open-source environment. Please consider exporting data in an open-source format.

7. Please use capital letters for LAURDAN, as it is an acronym.

8. JC-1 undergoes spectral shifts, so why use ratio metric measurement? It might be more appropriate to use the GP function for its evaluation.

© 2024. Published by The Company of Biologists under the terms of the Creative Commons Attribution License (https://creativecommons.org/licenses/by/4.0/).

9. How do the authors handle channel crosstalk? While they collect the full spectra, there is no demixing process for signals mentioned. Therefore, I assume they are adding the channels from the range of one fluorophore emission. Is this correct? It's not clear in the manuscript.

10. There is no figure with a scale bar or dimension information. Figures use color codes that hinder accessibility for color-blind readers. It is advisable to use grayscale for each channel and only use color in merged images (I suggest to follow new LUT recommendation, for instance avoid green and red).

Additionally, there is no numeric scale provided for the GP images.

11. The manuscript claims multi-dimensional capability for the VISION software, but there are no 3D or 4D examples provided to demonstrate its full potential.

12. There is no depiction of the GUI (Graphical User Interface). It's important to showcase the interface of the new software.

13. In the experimental section, there is missing information on microscopy parameters, such as dichroic mirrors used, laser power in quantitative units (not in %), pixel size, etc.

14. Regarding the dichroic mirror used, how much can its choice affect measurements when multiple excitation lines are needed? For instance, in cells with Pro12A and JC-1?

Reviewer 3

Advance summary and potential significance to field

The authors have developed a versatile open source software package for quantitative analysis of fluorescence intensity profiles of the surrounding membranes of lipid vesicles and cells, as well as the interior of such vesicles and cell cytoplasm. The software package combines image segmentation with a freely programmable mathematical operations on the intensity profiles of segmented areas.

In the manuscript, the authors present an overview of the functionality of their software package combined with several applications of their software package for analysis of biophysical parameters such as membrane phase separation membrane fluidity, mitochondrial health, and protein clustering. The application examples are well chosen for demonstrating the versatility and power of the developed software package, and as such suggests that the software package could indeed be of broad interest to the community for use in standardized analysis in related work.

Comments for the author

The manuscript is generally well written and concise, although requires a careful proof reading to correct spelling mistakes including in abstract of Phyton (sic) rather than Python.

Considering that the manuscript is a Tools and Resources submission, an important aspect is that the presented software package comes with a detailed manual including a troubleshooting section and preferably also a number of test images for future prospective users from the community. While the VISION software package and a software manual can be found at GitHub, there appears to be no trouble-shooting section for prospective users in particular related to the required image quality (i.e. signal-to-noise, projected pixel sampling, etc). As per GitHub repository, the authors further plan a set of YouTube tutorials on the use of the software. It is my opinion that the prompt availability of these tutorials in addition to the inclusion of a trouble-shooting section would provide the best guarantee that the developed VISION software package will potentially be broadly used by the user community. Thus I would urge the authors to complement the submission with these parts.

© 2024. Published by The Company of Biologists under the terms of the Creative Commons Attribution License (https://creativecommons.org/licenses/by/4.0/).

First revision

Author response to reviewers' comments

We also attached this letter as a formatted document for easier read.

Point-by-point reply to Reviewers comments.

We would like to express our gratitude to the Editor for considering our manuscript and the Reviewers for their valuable comments. We agree with all the points raised by the Reviewers and we implemented the suggestions made both in the manuscript and in the software. Furthermore, we implemented some additional features based on feedback we received from the scientific community since the software upload on Biorxiv. We believe that these changes improved both usability and applicability of our software. Below, we present a point-by-point reply to the Reviewers' comments, whereas changes in the manuscript were highlighted in yellow for easy tracking.

Reviewer 1 Comments for the author

Major points:

1. It is unclear in Figs. 4-7 the number of biological replicates that were performed to accumulate the n values presented. Please indicate the biological and technical replicates clearly in all figure legends.
As suggested by the Reviewer, we specified the number of technical and biological replicates in each figure's legend.

2. It is not clear that "lateral displacement" is "ΔX". This is in several figures, but only explained in the legend for Fig. 3. Could you explain this in all legends? Also, why is this in pixel units and not in nanometres, for example? That would help to get an idea as to the effect of vesicle size on the distance between membrane regions, or is this necessary to normalise data between different vesicle types? Is this somehow different in Fig. 7 where an approximate area was calculated from this value?
We added a sentence in the main text and in each figure's caption to clarify that ΔX refers to the lateral displacement. Furthermore, we modified the algorithm in order to return ΔX in µm units. As pointed by the Reviewer, this will help to potentially correlate changes in GP with vesicle sizes. Regarding the Figure 7, the areas indicated by black boxes do not refer to the integrated areas used for the profiling, but it rather identifies different regions along the membrane to help the reader appreciate the enhancement in contrast between regions with different membrane fluidity.

3. Given your conclusion in the "Analysis of phase separated vesicles" section (page 8) is that the DCA had no effect on ordered phases, but further increased the fluidity of the ordered phases, could you compare the binned values from the "GP_below" and "GP_above" in Fig. 4E & 4F? As I understand it, there should be a significant difference between the "GP_below" values in 4E & 4F, but not between the "GP_above", as they should correspond roughly to the disordered and ordered membrane regions respectively. Can you also further comment on the increase in disordered regions, as the reference you give (Zhou, et al., 2013, JBC) used a system where the membrane order could not change. Do you think that you are seeing an additional effect of DCA here in decreasing the fraction of ordered membrane regions?
As suggested by the Reviewer, we combined panel E and F in Figure 4 and directly compared values from the two GP sets with and without the bile acid. As already anticipated by the Reviewer, the combined plot confirmed the difference in GP values to be statistically significant for "GP_below" subsets with and w/o DCA, but not for the "GP_above" subsets. Thus, the combined panel better conveys the conclusion that the bile acid DCA further decreases the fluidity of membrane disordered phases but has no effect on the ordered ones. Regarding the results from literature (Zhou, et al., 2013, JBC), in their manuscript the authors concluded that bile acids stabilize phase separation in GPMVs by quantifying the percentage of phase-separated vesicles as a function of temperature and bile acid concentration. However, they did not quantify the change in membrane area of one phase over the other. Thus, although we cannot directly compare our observations

© 2024. Published by The Company of Biologists under the terms of the Creative Commons Attribution License (https://creativecommons.org/licenses/by/4.0/).

(i.e., increase in membrane disordered phase) with theirs, we can affirm that VISION allows for such quantification thanks to the module for membrane profiling.

4. Fig. S3 assessing the spectral spillover between the JC-1 and Pro12A probes is necessary, but performed in a way that doesn't properly assess the spillover of either probe. To assess the bleed-through of Pro12A into the JC-1 channels, cells should be treated with Pro12A or JC-1 alone and assessed using the same excitation and emission channels as would be used for the co-stained cells. The bleed-through from this control experiment should then be presented in Fig. S3 to show the bleed-through of Pro12A into the JC-1 emission channels after excitation with the 405 and 488 nm laser lines. The same should be done in the JC-1-stained cells to assess bleed-through into the Pro12A channels from both 405 and 488 excitation.
Following the Reviewer's suggestion, we reassessed the reciprocal spectral spillover of Pro12A and JC-1 dyes. Thus, in Figure S3 we show the results from images acquired from either unstained (for autofluorescence background) or single-stained cells using the same settings for co-stained experiments. The results confirmed negligible bleed-through of each dye into one another.

Minor points:

1. "Python" is misspelled in the abstract and should be corrected from "Phyton". There are a couple of other spelling mistakes and small grammatical errors in the manuscript that should also be corrected to enhance an otherwise clear and logical manuscript text.
As suggested by the Reviewer, we proofread the manuscript and corrected the typo and small grammatical errors.

2. The "(-)" and "(+)" are not very clear for the DCA treatments. Could you instead have "(-)DCA" and (+)DCA"?
As suggested by the Reviewers, we replaced "(-)" and "(+)" in Figure 4 with "w/o DCA" and "with DCA", respectively.

3. The details of the statistical analysis given in the legend for Fig. 4 (and in the "Statistical analysis" section of the methods) are incomplete. Please indicate the specifics of the "non-parametric t-test performed". For example, was it a Mann-Whitney or Kolmogorov-Smirnov test?
As suggested by the Reviewer, both in the legend of Figure 4 and in the "Statistical analysis" section we clarified the method used for assessment of statistical significance, namely the Mann-Whitney non-parametric t-test and the non-parametric one-way ANOVA using the Kruskal-Wallis test for GUVs and GMPVs analysis, respectively.

4. When referring to the lack of change of vesicle morphology after addition of DCA (page 8), the reference to Fig. 4A-C should be corrected to Fig. 4A & 4B. Fig. 4C refers to the GP and not the size/morphology, which you discuss in the next sentence.
We corrected this in the main text.

5. The fluorescence spectrum of several dyes, including Pro12A, is modified based on the solvent used to solubilise the dye. Could you please provide the details for the solvents used and the suppliers for Pro12A, JC-1, NR12A and NR12S?
Following the Reviewer's suggestion, we added the information regarding the probes' suppliers and solvents used in the section "Labelling of membranes and mitochondria".

*****

Reviewer 2 Comments for the author

1. At the outset of the manuscript, the authors missed an opportunity to credit the original work that proposed the GP function, opting instead for their own literature. This omission extends to other methods, like spectral phasor plots or GUV electroformation. I strongly recommend referencing the original works of Parasassi et al. (1991) for GP, Fereidouni (2012) for Spectral phasor plots, and Angelova (1986).
We addressed the Reviewer's suggestion and included the reference to the literature suggested. Specifically, we replaced the reference "Yu, W. et al. (1996) 'Fluorescence generalized polarization of cell membranes: a two-photon scanning microscopy approach.', Biophysical Journal,

© 2024. Published by The Company of Biologists under the terms of the Creative Commons Attribution License (https://creativecommons.org/licenses/by/4.0/).

70(2), p. 626. Available at: https://doi.org/10.1016/S0006-3495(96)79646-7” with the reference “Parasassi, T. et al. (1990) ‘Phase fluctuation in phospholipid membranes revealed by Laurdan fluorescence.’, Biophysical Journal, 57(6), pp. 1179–1186”, which introduced for the first time the equation for the Generalized Polarization (GP) calculation. Similarly, in the section “Software overview” we included the reference to the article “Fereidouni, F., Bader, A.N. and Gerritsen, H.C. (2012) ‘Spectral phasor analysis allows rapid and reliable unmixing of fluorescence microscopy spectral images’, Optics Express, 20(12), pp. 12729–12741. Available at: https://doi.org/10.1364/OE.20.012729”, which described the first example of spectral phasor analysis. Lastly, in the section “Preparation of synthetic vesicles” we added the reference to the article “Angelova, M.I. and Dimitrov, D.S. (1986) ‘Liposome electroformation’, Faraday Discussions of the Chemical Society, 81(0), pp. 303–311. Available at: https://doi.org/10.1039/DC9868100303”, which first described the preparation of GUVs via electroformation.

2. While the authors claim support for several file formats, only Zeiss formats (czi, lsm) or OME-tiff are guaranteed. I suggest tempering this claim or devising protocols to open files from Leica, Olympus, or Nikon.
Following the Reviewer’s suggestion, in addition to .lsm and .czi (Zeiss) formats, we implemented the .lif (Leica) and .nd2 (Nikon) formats. At this stage we were not able to implement other formats since we did not have access to the required instrumentation. Nevertheless, as stated in the GitHub repository, we are more than willing to implement additional formats upon the user’s request. Furthermore, in one of the tutorial YouTube videos, we describe how to easily analyse images in any of the non-implemented formats via previous conversion into .ome.tiff with Fiji. https://www.youtube.com/watch?v=NmAVgJb-E-g

3. Please provide a description and mathematical notation for the b-values calculated in the paper. It wasn’t clear until late in the manuscript which b-values were used, and there was no description of the mathematics involved.
Following the Reviewer’s suggestions we clarified which b-value we refer to in each of the examples on biological application given (text highlighted in yellow). We also described the equation for calculation of GP in the Supporting Information file.

4. The use of spectral phasor plot representation and analysis on b-value histograms is confusing. It seems like a backward use of a model-free method. First, a b-value is calculated with a mathematical model, and then the results are represented using a model-free approach. This is particularly puzzling for the GP, given the existing literature on spectral phasor analysis of LAURDAN fluorescence.
In the manuscript we present a phasor plot only in the software’s workflow schematic (Figure 1), as one of the many features of our software VISION. However, all analyses in the paper were conducted by calculating the actual generalized polarization according to the given formula (or intensity ratios for JC-1), and not by performing spectral phasor analysis.

5. There is no mention of other software used in the field for spectroscopy on images. Some examples include SimFCS, Spectral image analysis (Fiji module), Spectral phasor Fiji plugin, and PhasorPy for phasor analysis of FLIM and HSI data. It’s advisable to acknowledge them.
Following the Reviewer’s comment, we provided references for the software and plugins for spectral phasor analysis (highlighted in the main text), so that they could be easily accessed by the reader.

6. What are the file formats for exporting the b-value images generated? This is not mentioned in the manuscript. Also, exporting data to an Excel spreadsheet in a proprietary format is not ideal for working in an open-source environment. Please consider exporting data in an open-source format.
In the section “Data Exporting” we clarified that the user could choose to export the ß--colour coded image in .tiff. Furthermore, based on the Reviewer’s suggestion we implemented in VISION (and stated in the main text´, within the same section) the possibility to export the results both in .csv and .xlsl. We also made possible to export the metadata (i.e., parameters used for masking and image settings) in .json format.

7. Please use capital letters for LAURDAN, as it is an acronym.
We replaced the word “Laurdan” with “LAURDAN” as suggested by the Reviewer.

© 2024. Published by The Company of Biologists under the terms of the Creative Commons Attribution License (https://creativecommons.org/licenses/by/4.0/).

8. JC-1 undergoes spectral shifts, so why use ratiometric measurement? It might be more appropriate to use the GP function for its evaluation.
JC-1 can exist in two distinct forms, either as monomers or aggregates (healthy mitochondria). Each of this state is characterized by its own distinct emission spectrum. Typically, the ratio between the green and red channels has been calculated in the literature, as it is straightforward and can be performed both in microscopy and flow cytometry settings. Therefore, we decided to remain consistent with the well-established approach of calculating the ratio, so to allow for direct comparison of our results with those in literature.

9. How do the authors handle channel crosstalk? While they collect the full spectra, there is no demixing process for signals mentioned. Therefore, I assume they are adding the channels from the range of one fluorophore emission. Is this correct? It's not clear in the manuscript.
As suggested by the Reviewer, we clarified the approach we followed to calculate the GP values and ratio for JC-1 in the Methods section. Briefly, for spectral images we collected the full emission spectrum, but we used only two channels from the n recorded. Indeed, we did not perform any demixing operation, but recurred to the full emission spectrum to select the best pair of channels (those that yielded the highest resolution) for GP or monomers/aggregates ratio calculation. Furthermore, in the biological examples provided, we either used one probe at a time or combined Pro12A with JC-1, which have negligible spectral overlap (i.e., spillover, as we show in Fig. S3) and are suitable to measure both properties (i.e., membrane fluidity and mitochondria membrane potential) at once.

10. There is no figure with a scale bar or dimension information. Figures use colour codes that hinder accessibility for colour-blind readers. It is advisable to use grayscale for each channel and only use colour in merged images (I suggest to follow new LUT recommendation, for instance avoid green and red). Additionally, there is no numeric scale provided for the GP images.
Following the Reviewer's suggestion, we added a scale bar in each microscopy image and specified its dimension and units in the figure's captions. Furthermore, we modified the colour scheme in each figure according to the LUT recommendations to improve readability for colour-blinded readers. Finally, we also added GP numeric scales in corresponding figures.

11. The manuscript claims multi-dimensional capability for the VISION software, but there are no 3D or 4D examples provided to demonstrate its full potential.
Based on the Reviewer's suggestion, we presented an example of analysis of multidimensional images in the tutorial video for VISION that we provided together with the software. There, we describe each step required to perform manual or automatized analysis of multidimensional stacks.

12. There is no depiction of the GUI (Graphical User Interface). It's important to showcase the interface of the new software.
Based on the Reviewer's comment, we provided several screenshots of the GUI for VISION in the Supporting Information, where we show what the user would see during each phase of the image analysis, from the masking of raw image to the analysis of individual objects. Moreover, we now provide a YouTube tutorial that covers all the details of the GUI.

13. In the experimental section, there is missing information on microscopy parameters, such as dichroic mirrors used, laser power in quantitative units (not in %), pixel size, etc.
Following the Reviewer's suggestion, we added a detailed description of the microscopy parameters used in the section "Confocal and STED imaging".

14. Regarding the dichroic mirror used, how much can its choice affect measurements when multiple excitation lines are needed? For instance, in cells with Pro12A and JC-1?
As described in the experimental section, we performed sequential acquisition of the signal derived from the plasma and mitochondrial membrane. Thus, we optimized microscopy settings (i.e., dichroic mirrors and acquisition ranges) in order to avoid any spectral overlap between the two measurements (see also SI). Under these conditions, GP estimation is robust for different dichroic mirrors and excitation schemes.

*****
Reviewer 3 Comments for the author

© 2024. Published by The Company of Biologists under the terms of the Creative Commons Attribution License (https://creativecommons.org/licenses/by/4.0/).

1. The manuscript is generally well written and concise, although requires a careful proof reading to correct spelling mistakes including in abstract of Phyton (sic) rather than Python.
We performed a thorough proofreading and corrected the typos.

2. Considering that the manuscript is a Tools and Resources submission, an important aspect is that the presented software package comes with a detailed manual including a troubleshooting section and preferably also a number of test images for future prospective users from the community. While the VISION software package and a software manual can be found at GitHub, there appears to be no troubleshooting section for prospective users in particular related to the required image quality (i.e. signal-to-noise, projected pixel sampling, etc).
As suggested by the Reviewer, we provided a detailed guide for the use of VISION software together with a troubleshooting section. The user can easily access both contents via the link to the GitHub repository.
https://github.com/biosciflo/VISION

3. As per GitHub repository, the authors further plan a set of YouTube tutorials on the use of the software. It is my opinion that the prompt availability of these tutorials in addition to the inclusion of a trouble-shooting section would provide the best guarantee that the developed VISION software package will potentially be broadly used by the user community. Thus, I would urge the authors to complement the submission with these parts.
As suggested by the Reviewer, we produced several tutorial YouTube videos whose links are provided in the GitHub repository and on the manuscript. In these tutorials, we describe in detail the functionalities of the VISION software and give some examples on how to achieve optimal image analysis in VISION.
https://www.youtube.com/watch?v=ZDZju8mgNiY
https://www.youtube.com/watch?v=NmAVgJb-E-g

Second decision letter

MS ID#: JOCES/2024/262166

MS TITLE: VISION - an open-source software for automated multi-dimensional image analysis of cellular biophysics

AUTHORS: Florian Weber, Sofiia Iskrak, Franziska Ragaller, Jan Schlegel, Birgit Plochberger, Erdinc Sezgin, and Luca Andronico
ARTICLE TYPE: Tools and Resources

I am happy to tell you that your manuscript has been accepted for publication in Journal of Cell Science, pending standard publication integrity checks.

Reviewer 1

Advance summary and potential significance to field

In the manuscript by Weber, F. and colleagues, the authors present an open-source software package, VISION, for automated segmentation and analysis of multidimensional images. They then go on to demonstrate its application for assessment of probes for membrane fluidity, mitochondrial health and individual protein localisation, confirming several findings from previous studies, while also expanding on these findings through the multidimensional analysis. Overall, the study is well presented, and the software is likely to provide a powerful tool to microscopists, particularly with the automated segmentation that they have implemented within the platform.

Comments for the author

© 2024. Published by The Company of Biologists under the terms of the Creative Commons Attribution License (https://creativecommons.org/licenses/by/4.0/).

The authors have satisfactorily addressed all of my comments and I now believe that the manuscript is ready for publication.

Reviewer 2

Advance summary and potential significance to field

VISION software enables biologists to analyze different image data sets with sophisticated analysis, an easy interface, and an accurate report. This is a valuable resource for the community.

Comments for the author

The new manuscript significantly improves on the first version. I am happy to support its publication.

Reviewer 3

Advance summary and potential significance to field

The authors have developed a versatile open source software package for quantitative analysis of fluorescence intensity profiles of the surrounding membranes of lipid vesicles and cells, as well as the interior of such vesicles and cell cytoplasm. The software package combines image segmentation with a freely programmable mathematical operations on the intensity profiles of segmented areas.

In the manuscript, the authors present an overview of the functionality of their software package combined with several applications of their software package for analysis of biophysical parameters such as membrane phase separation membrane fluidity, mitochondrial health, and protein clustering. The application examples are well chosen for demonstrating the versatility and power of the developed software package, and as such suggests that the software package could indeed be of broad interest to the community for use in standardized analysis in related work. Furthermore, the authors have now complemented the manuscript with a set of online tutorials that demonstrate step-by-step workflow examples of use of the VISION software.

Comments for the author

I am satisfied with the revisions that have been made to address the previous reviewers comments. I expect that the included video tutorials will help with adaptation of the VISION software for analysis by other researchers thus meeting the key aim of the manuscript itself.

© 2024. Published by The Company of Biologists under the terms of the Creative Commons Attribution License (https://creativecommons.org/licenses/by/4.0/).
